# Fluid deresuscitation in critically ill children: comparing perspectives of intensivists and nephrologists

**DOI:** 10.3389/fped.2024.1484893

**Published:** 2024-10-28

**Authors:** Chloe G. Braun, David J. Askenazi, Javier A. Neyra, Priya Prabhakaran, A. K. M. Fazlur Rahman, Tennille N. Webb, James D. Odum

**Affiliations:** ^1^ Division of Pediatric Critical Care, Department of Pediatrics, Heersink School of Medicine, The University of Alabama at Birmingham, Birmingham, AL, United States; ^2^ Division of Nephrology, Department of Pediatrics, Heersink School of Medicine, The University of Alabama at Birmingham, Birmingham, AL, United States; ^3^ Division of Nephrology, Department of Medicine, Heersink School of Medicine, The University of Alabama at Birmingham, Birmingham, AL, United States; ^4^ Department of Biostatistics, University of Alabama at Birmingham, Birmingham, AL, United States

**Keywords:** fluid accumulation, deresuscitation, critical care, nephrology, CRRT—continuous renal replacement therapy

## Abstract

**Introduction:**

Fluid accumulation, presently defined as a pathologic state of overhydration/volume overload associated with clinical impact, is common and associated with worse outcomes. At times, deresuscitation, the active removal of fluid via diuretics or ultrafiltration, is necessary. There is no consensus regarding deresuscitation in children admitted to the pediatric intensive care unit. Little is known regarding perceptions and practices among pediatric intensivists and nephrologists regarding fluid provision and deresuscitation.

**Methods:**

Cross-sectional electronic survey of pediatric nephrologists and intensivists from academic societies in the United States designed to better understand fluid management between disciplines. A clinical vignette was used to characterize the perceptions of optimal timing and method of deresuscitation initiation at four timepoints that correspond to different stages of shock.

**Results:**

In total, 179 respondents (140 intensivists, 39 nephrologists) completed the survey. Most 75.4% (135/179) providers believe discussing fluid balance and initiating fluid deresuscitation in pediatric intensive care unit (PICU) patients is “very important”. The first clinical vignette time point (corresponding to resuscitation phase of early shock) had the most dissimilarity between intensivists and nephrologists (*p* = 0.01) with regards to initiation of deresuscitation. However, providers demonstrated increasing agreement in their responses to initiate deresuscitation as the clinical vignette progressed. Compared to intensivists, nephrologists were more likely to choose “dialysis or ultrafiltration” as a deresuscitation method during the optimization [10.3 vs. 2.9% (*p* = 0.07)], stabilization [18.0% vs. 3.6% (*p* < 0.01)], and evacuation [48.7% vs. 23.6% (*p* < 0.01)] phases of shock. Conversely, intensivists were more likely to utilize scheduled diuretics than nephrologists [47.1% vs. 28.2% (*p* = 0.04)] later on in the patient course.

**Discussion:**

Most physicians believe that discussing fluid balance and deresuscitation is important. Nevertheless, when to initiate deresuscitation and how to accomplish it differed between nephrologist and intensivists. Widely understood and operationalizable definitions, further research, and eventually evidence-based guidelines are needed to help guide care.

## 1 Introduction

Intravenous fluids (IVF) are ubiquitously administered in the pediatric intensive care unit (PICU) with the physiologically derived goals of improving cardiac output, meeting daily nutrition needs, and delivering medications/blood products. Unfortunately, this common practice can result in fluid accumulation—a pathologic state of overhydration/volume overload, associated with clinical impact which may vary by age, comorbidity, and phase of illness (1). Some authors utilize the term fluid overload interchangeably with fluid accumulation to describe a pathologic state of positive fluid balance associated with adverse events (2, 3). Excessive fluid results in interstitial edema, which by a variety of mechanisms (impaired oxygen and metabolite diffusion, impaired capillary blood flow, disturbed tissue architecture etc.) can contribute to organ failure (1, 4). It should be noted that patients can have fluid accumulation while still being intravascularly hypovolemic due to increase in total body water. Fluid accumulation is associated with poor outcomes, including increased risk for mortality, increased length of mechanical ventilation, increased hospital and PICU lengths of stay, and increased use of continuous renal replacement therapy (CRRT) (5–12).

**Figure 1:**
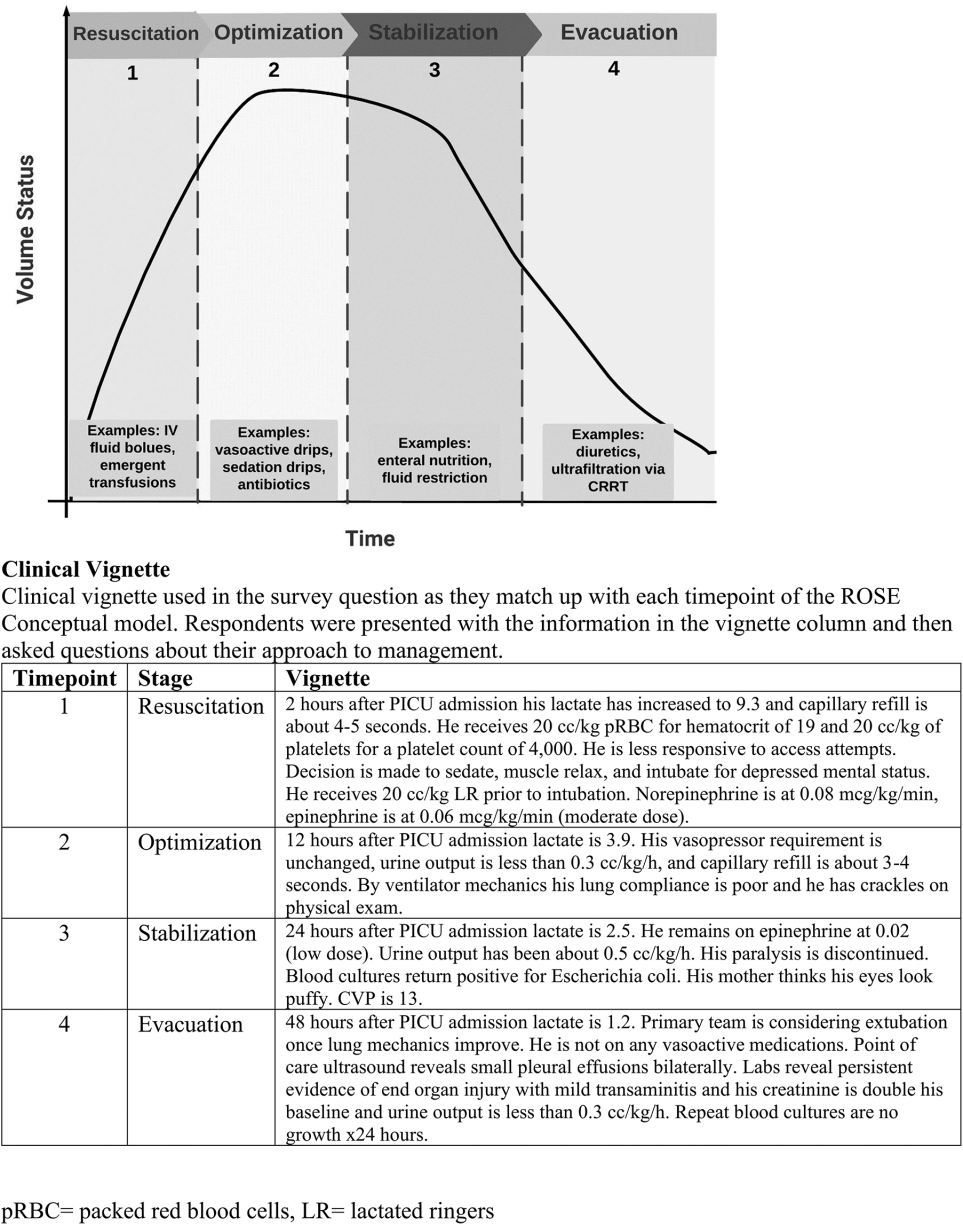
ROSE diagram. ROSE diagram that illustrates a conceptual framework of fluid management in shock. A patient's volume status ’s plotted over the clinical course. (Adapted from Malbrain, 2024).

One of the most common scenarios among PICU patients that leads to fluid accumulation is shock. While the incidence of shock among critically ill pediatric patients varies in the literature, it is one of the leading causes of morbidity and mortality (13). Characterized by inadequate oxygen and glucose delivery to meet metabolic demand, patients with shock commonly receive administration of fluids in the form of boluses, or continuous administration with the goal to improve oxygen delivery to vital tissues. The “ROSE” conceptual model— resuscitation, optimization, stabilization, evacuation—is a framework that describes four distinct phases of fluid management in shock (14) (Figure 1). Resuscitation (**R**) is the initial phase of shock, where a patient is at high risk for mortality if oxygen delivery is not improved. **R** prioritizes organ rescue and often includes aggressive fluid administration (up to 60 ml/kg) (15). As a result of damaged vascular endothelium and the endothelial glycocalyx (most common in septic shock), fluid tends to extravasate from the intravascular space, thus fluid initially deemed helpful can become problematic (16). The second stage, optimization (**O**) relies on careful titration of fluid administration, optimizing cardiac output, and minimizing additional organ injury by congestion and edema (17). Third, stabilization (**S**) focuses on organ support as the patient slowly returns to homeostasis. In the stabilization phase, intensivists focus on late conservative fluid management and commonly target an even or net negative fluid balance, which has been associated with decreased mortality (18). Finally, the evacuation (**E**) phase emphasizes organ recovery and includes strategies to return to a neutral fluid balance. The evacuation phase classically has included deresuscitation—presently defined as the active removal of fluid via ultrafiltration or diuretics (19). Other authors have used the following definition: active fluid removal to treat fluid accumulation causing organ dysfunction (1). Notably, deresuscitation involves more than fluid restriction.

While consensus-based guidelines offer more concrete recommendations on appropriate resuscitation of the critically ill child in shock, consensus guidelines for timing or method of fluid deresuscitation do not currently exist (15). In critically ill patients, deresuscitation involves close communication between intensivists and nephrologists, especially when CRRT is being considered for patients with acute kidney injury, significant fluid accumulation, or electrolyte derangements (20). Whether differences between pediatric nephrologist and intensivists exist in the perceived best strategies to prevent and treat fluid accumulation has not been fully evaluated.

In order to improve our understanding of providers’ perceptions of fluid accumulation and subsequently deresuscitation, we performed a survey-based study anchored on a clinical vignette. We hypothesized that differences exist between pediatric intensivists and pediatric nephrologists regarding (a) how providers determine the transition point between resuscitation and deresuscitation and (b) what strategies providers use to mitigate and correct fluid accumulation.

## 2 Methods

### 2.1 Study population

We performed a cross-sectional survey-based study to assess perceptions and approaches to pediatric patients with fluid accumulation. We surveyed attending physicians in pediatric nephrology and pediatric critical care as they are the two groups most closely involved in evaluating fluid status and managing the consequences of fluid accumulation in the PICU population. The surveyed population represents academic clinicians in majority US-based hospitals and utilized convenience-based sampling by disseminating through the Pediatric Society of Critical Care Medicine website and the following listservs: pediatric Continuous Renal Replacement Therapy (pCRRT), Pediatric Acute Lung Injury and Sepsis Investigators Network (PALISI), and American Society of Pediatric Nephrology (ASPN). A nonresponse rate was not calculated because the total sample size was unknown. This is due in part to inability to quantify number of providers targeted because of duplicate email addresses included in the listservs. According to the American Board of Pediatrics, 729 pediatricians currently maintain Pediatric Nephrology certification, while 3,128 maintain certification in Pediatric Critical Care Medicine (21).

### 2.2 Survey design

The survey was 25 questions long and took less than 10 min to complete (Supplementary File S1). It was generated using structured questions intended to discern provider perceptions regarding deresuscitation. The initial survey questions provided respondents an opportunity to share their perspectives on fluid accumulation, deresuscitation, and the clinical culture at their institution. A 5-point Likert scale was utilized when possible. A clinical vignetter was designed with four time points that align with the ROSE framework (Resuscitation, Optimization, Stabilization, and Evacuation) for fluid management in shock (Figure 1). It outlined the clinical course of a 4-year-old patient with leukemia presenting in shock (heart rate 140 beats per minute, blood pressure 68/39 mmHg, respiratory rate 26 breaths per minute, temperature 103°F, lactate 7.0 mmol/L) who received 3 × 20 ml/kg fluid boluses (lactated ringers) and was placed on catecholamine infusions to maintain mean arterial blood pressure > 10th percentile for age. Following this vignette introduction, respondents were given follow-up details as the case progressed and then asked to decide whether they believed the patient was still in shock; whether initiation of deresuscitation was appropriate; and if so, what strategies they would utilize to perform deresuscitation. The survey concluded by asking demographic information about the provider (e.g., total years in practice) and their institution (e.g., number of PICU beds).

The survey was anonymous, voluntary, and responses were confidential. Consent from the provider, mentioned at the beginning of the survey, was assumed upon completion. Since this survey was novel and designed by our study team, no current validity evidence exists in the literature. To address internal consistency, the survey was independently pre-tested and reviewed by 2 expert pediatric intensivists and 2 expert pediatric nephrologists. After reaching agreement through iterative review, the survey was entered into REDCap which served as the end-user survey interface and the data management platform (20, 21). Data was stored within REDCap study database and on password protected institutional devices. Data collection began in November 2023 and concluded in May 2024. We were unable to control for respondents filling out the survey multiple times as we did not collect any personal identifiers. The institutional review board for the University of Alabama at Birmingham approved this study (IRB-300011177).

### 2.3 Statistical analysis

All responses were mandatory, so we did not have to account for missing data. Descriptive analyses (means (standard deviations), medians (interquartile ranges), and frequency distributions (%)) described study participants and relevant characteristics. Categorical responses were summarized as frequency (%) and compared between groups using Chi-square or Fischer exact test. Proportion difference with 95% confidence interval were reported. Continuous measures were compared between groups using *t*-test or Wilcoxon rank-sum tests as appropriate. Similar analyses were performed for the subgroups—whether responses differ by years of experience or the size of the institution within each group as well as overall. For years of experience, we chose 7 years as a cut-off as this was the median number of years post-training for intensivists. We chose number of ICU beds as a surrogate for size of institution and opted for a cut-off of 26 beds as this was the median for both intensivists’ and nephrologists’ responses. All statistical tests were two-tailed with *p* value <0.05 used to indicate statistical significance. Analyses were performed using SAS version 9.4 software (SAS Institute, Inc.; Cary, NC).

## 3 Results

### 3.1 Demographics

The target study population of this survey was pediatric intensivists (including cardiac intensivists but excluding neonatologists) and pediatric nephrologists who have finished training. The survey was completed by 140 pediatric intensivists and 39 pediatric nephrologists for a total of 179 respondents. Only 4 providers opened but did not complete the survey (2.2%). The median (IQR) number of years post-medical training was 7 (4, 15) for intensivists and 10 (3, 19) for nephrologists (*p* = 0.24). Providers from both groups self-reported a median (IQR) of 26 (20, 40) PICU beds at their institution. Regarding post-graduate trainees at their institution, 95.0% of providers reported having pediatric residents, 79.9% reported having pediatric critical care fellows, and 55.9% reported pediatric nephrology fellows. CRRT capabilities were reported by 93.3% of survey respondents and extracorporeal membrane oxygenation capabilities were reported by 85.5% of providers (Table 1).

**Table 1:** Self-reported characteristics of survey respondents.

	Both groups	Intensivists	Nephrologists	*P* value
Number of respondents	179	140	39	
Median years post training (IQR)	8 (4, 16)	7 (4, 15)	10 (3, 19)	0.24
Median PICU beds (IQR)	26 (20, 24)	26 (20, 40)	26 (20, 40)	0.81
Post graduate trainees				
Pediatric residents	95.0%	95.7%	92.3%	0.41
Pediatric Critical Care fellows	79.9%	80.7%	76.9%	0.60
Pediatric Nephrology fellows	55.9%	55.7%	56.4%	0.94
Extracorporeal capabilities				
ECMO	85.5%	86.4%	82.1%	0.49
CRRT	93.9%	93.6%	97.4%	0.69
Peritoneal Dialysis	88.3%	87.1%	92.3%	0.58
Hemodialysis	89.9%	89.3%	92.3%	0.77
PIRRT	38.5%	35.0%	53.9%	0.07
TPE	83.2%	83.6%	82.1%	0.82

IQR, interquartile range; ECMO, extracorporeal membrane oxygenation; CRRT, continuous renal replacement therapy; PIRRT, prolonged intermitted renal replacement therapy; TPE, therapeutic plasma exchange.

### 3.2 Importance of fluid balance

The overall perspective regarding fluid balance was not significantly different between intensivists and nephrologists. At large, 135/179 (75.4%) of providers believed discussing fluid balance and initiating fluid deresuscitation in PICU patients is “very important”, but when asked how their institution views this practice only 79/179 (44.1%) responded “very important”. Fluid balance is explicitly discussed “all of the time” when rounding on critically ill patients by 56.0% of intensivists and 43.6% of nephrologists (*p* = 0.15). When asked if there is consistency among providers within the respondent's specialty in determining clinical stability prior to initiating deresuscitation, less than half of respondents agreed or strongly agreed that practice habits are consistent (strongly disagree 6.1%, disagree 29.6%, neither disagree nor agree 24.0%, agree 30.7%, and strongly agree 9.5%). Institutional deresuscitation protocols were rare with only 2.2% of providers reporting following one.

### 3.3 Measuring fluid balance

The majority of intensivists (70.0%) and nephrologists (84.6%) report using weight in addition to intake/output method to assess fluid balance. Ultrasonography to assess fluid balance was infrequent (intensivists 31.4% vs. nephrologists 18.0%, *p* = 0.11). Greater than 90% of providers from both groups selected each of the following as crucial components to their assessment of fluid balance: physical exam, urine output, intake and output, and daily weights (Supplementary Figure S1). The majority of providers (83.2%) stated they have the tools necessary to accurately assess and recommend fluid goals, but upon free text questioning common responses to factors limiting fluid balance assessment included inaccurate weights, inconsistently charted intake and output, and informal electronic medical record recording and ordering.

### 3.4 Deresuscitation initiation

A clinical vignette was used to characterize the perceptions of optimal timing and method of deresuscitation initiation. The first time point (corresponding to resuscitation) had the most dissimilarity between intensivists and nephrologists with nephrologists more likely to initiate deresuscitation [18.0% vs. 6.4% (*p* = 0.01)] (Table 2). More than half of all providers favored initiating deresuscitation by the second timepoint (optimization) which remained consistent throughout the remainder of the vignette. Nephrologists were more likely than intensivists to choose “Dialysis or ultrafiltration” for deresuscitation at time points 3 and 4 respectively (18.0% vs. 3.6% [*p* < 0.05], and 48.7% vs. 23.6% [*p* < 0.01]). This pattern persisted for timepoint 2, though did not reach statistical significance 10.3% vs. 2.9% [*p* = 0.07]. Selection of specific fluid management (e.g., decreasing maintenance IVF) or diuretic management (e.g., infusion of loop diuretic) was similar between groups with the exception of the fourth time point where intensivists were more likely to select intermittent loop diuretic use [47.1% vs. 28.2% (*p* = 0.04)] (Table 3). Within specialties there was inconsistency among respondents when it came to method for deresuscitation and very few methods were selected by a majority of providers.

**Table 2:** Deresuscitation initiation.

Answer option	Intensivists	Nephrologists	*P* value
Case time point 1: Resuscitation phase			
Patient is in shock Deresuscitation is appropriate	6.4% (9/140)	18.0% (7/39)	0.01
Patient is in shock Deresuscitation is inappropriate	92.9% (130/140)	76.9% (30/39)	
Patient is not in shock Deresuscitation is appropriate	0%	0%	
Patient is not in shock Deresuscitation is inappropriate	0.7% (1/140)	5.1% (2/39)	
Case time point 2: Optimization phase			
Patient is in shock Deresuscitation is appropriate	56.4% (79/140)	51.3% (20/39)	0.06
Patient is in shock Deresuscitation is inappropriate	26.4% (37/140)	15.3% (6/39)	
Patient is not in shock Deresuscitation is appropriate	7.9% (11/140)	23.1% (9/39)	
Patient is not in shock Deresuscitation is inappropriate	9.3% (13/140)	10.3% (4/39)	
Case time point 3: Stabilization phase			
Patient is in shock Deresuscitation is appropriate	33.6% (47/140)	25.6% (10/39)	0.37
Patient is in shock Deresuscitation is inappropriate	1.4% (2/140)	5.1% (2/39)	
Patient is not in shock Deresuscitation is appropriate	60.7% (85/140)	66.7% (26/39)	
Patient is not in shock Deresuscitation is inappropriate	4.3% (6/140)	2.6% (1/39)	
Case timepoint 4: Evacuation phase			
Patient is in shock Deresuscitation is appropriate	1.4% (2/140)	0%	0.60
Patient is in shock Deresuscitation is inappropriate	0%	0%	
Patient is not in shock Deresuscitation is appropriate	94.3% (132/140)	100% (39/39)	
Patient is not in shock Deresuscitation is inappropriate	4.3% (6/140)	0%	

Comparison of intensivists’ to nephrologists’ responses to whether the clinical vignette patient is (1) in shock or not in shock and (2) initiation of deresuscitation is appropriate or inappropriate. Time points refer to each stage of ROSE framework. See Figure 1 for framework and case timepoint descriptions. Vignette timepoint 1, 2, 3, and 4 corresponds to the resuscitation phase, optimization phase, stabilization phase, and evacuation phase respectively.

**Table 3:** Preferred methods of deresuscitation at each clinical vignette timepoint.

Case time point #1 Resuscitation: Deresuscitation next step?	Intensivists	Nephrologists	*P* value
Stop providing fluid boluses	3.6%	18.0%	**<0** **.** **01**
Decrease (or discontinue) maintenance IV fluids	2.9%	7.7%	0.18
One time dose of loop diuretic (subsequent doses depending on response)	0.7%	2.6%	0.39
Intermittent loop diuretic scheduled	0%	5.1%	**<0**.**05**
Infusion of loop diuretic	0.7%	0%	1.0
Dialysis/ultrafiltration	0%	0%	1.0
Initial dose of loop diuretic followed by infusion	0.7%	0%	1.0
Other	1.4%	0%	0.37
Case time point #2 Optimization: Deresuscitation next step?			
Stop providing fluid boluses	49.3%	51.3%	0.86
Decrease (or discontinue) maintenance IV fluids	42.9%	59.0%	0.11
One time dose of loop diuretic (subsequent doses depending on response)	34.3%	53.9%	**0**.**04**
Intermittent loop diuretic scheduled	4.3%	7.7%	0.41
Infusion of loop diuretic	17.1%	12.8%	0.63
Dialysis/ultrafiltration	2.9%	10.3%	0.07
Initial dose of loop diuretic followed by infusion	8.6%	5.1%	0.74
Other	5.0%	0%	0.35
Case time point #3 Stabilization: Deresuscitation next step?			
Stop providing fluid boluses	70.7%	56.4%	0.09
Decrease (or discontinue) maintenance IV fluids	65.0%	53.9%	0.20
One time dose of loop diuretic (subsequent doses depending on response)	46.4%	38.5%	0.38
Intermittent loop diuretic scheduled	25.0%	23.1%	0.81
Infusion of loop diuretic	30.0%	30.8%	0.93
Dialysis/ultrafiltration	3.6%	18.0%	**<0**.**01**
Initial dose of loop diuretic followed by infusion	15.0%	7.7%	0.24
Other	5.0%	2.6%	1.0
Case time point #4 Evacuation: Deresuscitation next step?			
Stop providing fluid boluses	63.6%	60.0%	0.60
Decrease (or discontinue) maintenance IV fluids	59.3%	53.9%	0.54
One time dose of loop diuretic (subsequent doses depending on response)	22.9%	33.3%	0.18
Intermittent loop diuretic scheduled	47.1%	28.2%	**0**.**04**
Infusion of loop diuretic	31.4%	25.6%	0.49
Dialysis/ultrafiltration	23.6%	48.7%	**<0**.**01**
Initial dose of loop diuretic followed by infusion	17.1%	10.3%	0.30
Other	6.4%	5.1%	1.0

Participants were instructed to choose all that apply and could select multiple deresuscitation management strategies at each time point. Time points refer to each stage of ROSE framework. See Figure 1 for framework and timepoint descriptions.

Bold values represent statistically significant *p* values.

### 3.5 Subgroup analysis

*a priori* we determined provider level of experience (<7 years vs. ≥7 years) and the number of PICU beds at the respondents’ institution (<26 beds vs. ≥26 beds, as a surrogate for patient volume) as relevant factors that could impact a provider's response regarding if a patient was in shock and if deresuscitation was appropriate. In this subgroup analysis, a statistically significant difference between specialties was observed at time point 1 and 2 among providers practicing at institutions with ≥26 PICU beds (Supplementary Table S1), whereas no difference was seen at this time point in the full cohort, at institutions with <26 PICU beds, or when analyzed by years of experience (Supplementary Tables S1 and S2). We also looked at provider responses to these same questions within specialty groups to see if institution size or experience elucidated differences to practice approach. Supplementary Table S3 define nephrologists’ responses comparing institution size and years of experience. Supplementary Table S4 represent intensivists’ responses comparing institution size and years of experience. Within subspecialties, the subgroup analysis did not detect any differences in responses when separated by our cut offs.

## 4 Discussion

This cross-sectional survey of pediatric intensivists and nephrologists provides insight into providers’ perceptions and practices regarding fluid deresuscitation in critically ill children. As previously alluded to, fluid accumulation is associated with poor clinical outcomes in children admitted to the PICU (5–11, 22, 23). This negative association has been shown in a dose dependent manner (12) and remains important even early in a PICU course (24). We hypothesized there would be significant differences between intensivists’ and nephrologists’ responses regarding optimal timing and preferred strategies to initiate deresuscitation. Ultimately, we found that both groups agreed when it came to general fluid management principles. The respondents of this survey seem to acknowledge the importance of identifying and managing fluid accumulation as 75.4% of providers believed discussing fluid balance and initiating fluid deresuscitation in PICU patients is “very important”. Notably however, this response of “very important” falls to 44.1% when respondents were asked about their colleagues’ views on importance of fluid discussions suggesting room for improvement. Regarding specifics of initiating deresuscitation there was less consistency across providers. Nephrologists deemed patients ready for ready for deresuscitation earlier in the course than intensivists, however this difference narrowed at later case time points.

Understanding what contributes to fluid accumulation may assist in modifying it. Fluid boluses are an important component of the resuscitation phase; however, volume of maintenance and replacement IVF have been shown to significantly exceed total volume of resuscitation fluids. Fluids for resuscitation, maintenance, and replacement account for a smaller proportion of cumulative fluid balance than fluid creep—unintentional volume administered as a vehicle for other medications or electrolytes (25). A retrospective study in pediatric cardiac surgical patients concluded that patients with greater fluid creep had greater odds of cumulative fluid balance exceeding 10% and mortality (26). From our survey, the most commonly identified steps during the resuscitation phase that could limit positive fluid balance were a reduction in boluses or decrease maintenance fluids. For the remaining three case points, these choices continued to be the top 2 most selected management options. While limiting boluses or decreasing maintenance fluids are potentially modifiable contributors to fluid accumulation, adult data reveals fluid restriction alone has limited efficacy in preventing mortality (27–29).

In addition to limiting total fluid in, diuretic therapy can be utilized when initiating deresuscitation. Our survey identified that nephrologists were more likely to introduce diuretics early in the patient course, while intensivists were more likely to utilize diuretics later in the course. We also identified inconsistencies regarding approaches to diuretics (ex. infusion vs. intermittent dosing) and few options reached a majority. Responses related to diuretic options exceeded 100%, which indicates some providers selected multiple diuretic based options. This insinuates the approach to diuretic prescription is nuanced and highlights the lack of guidance in the literature. In one study, PICU patients who receive furosemide were less likely to develop a cumulative fluid balance >5%, but there is a paucity of pediatric literature about optimal timing of diuretic initiation and safety of doing so while still in shock (30).

One reason nephrologists may have been less likely to select diuretic use during the evacuation phase could be that they were more likely to select ultrafiltration (48.7% vs. 23.6%). Notably, nephrologists’ preference for ultrafiltration exceeded intensivists’ in the optimization and stabilization phase as well. This may be explained by differences in specialized training—nephrologists being more familiarized with prescription of ultrafiltration vs. intensivists being more familiar with technical skills/sedation required for dialysis catheter placement. Future work should focus on factors that intensivists and nephrologists weigh in deciding between diuretics and ultrafiltration.

Like diuretic administration, the pediatric literature guiding CRRT initiation for fluid accumulation is lacking and limited to retrospective or observational data (20). Prior randomized control trials in adults that use thresholds of serum creatinine suggest neither accelerated nor postponed CRRT initiation is best (31, 32). Historically in pediatrics, CRRT initiation has been based off severity of fluid accumulation (33) though an evidence-based threshold does not exist (20). There is observational data that reveal significant increase in mortality if CRRT is initiated at 20% positive cumulative fluid balance compared to 10% (34). TAKING FOCUS 2 recently described a clinical decision support algorithm to help guide CRRT initiation which found that CRRT days and rates of mortality decreased after algorithm implementation (33).

Still, it stands there are no consensus-based guidelines for how and when to perform deresuscitation which is reinforced in our survey as only 2.2% of providers report utilizing an institutional protocol. Complicating this matter is that identifying fluid imbalance has innate inaccuracies that our survey briefly addressed by some free text answers. Malbrain et al. suggests deresuscitation is appropriate once “salvage resuscitation” is finished—but the specifics of what defines salvage resuscitation is imprecise (17). The Pediatric Acute Disease Quality Initiative collaborators identified that rigorous science dedicated to managing fluid balance and fluid accumulation are lacking and recommended a working group dedicated to better understanding fluid balance (3). Notably, deresuscitation protocols have been shown to be feasible with potential clinical benefits in adults, yet a significant gap remains in implementing standardized deresuscitation practices in pediatrics (35, 36).

Our study is unique in including both intensivists and nephrologists, who frequently collaborate in caring for critically ill patients with acute kidney injury and fluid accumulation. However, this study has a few notable limitations. Innate to survey-based studies, the results represent providers’ perceptions of their practice, not their actual practice patterns. It is vulnerable to nonresponse bias, measurement bias, and coverage bias. We were also unable to control for respondents filling out multiple times. We think our survey was brief with high yield questions, thus limiting incomplete survey responses. There were only 4 providers who started but did not finish the survey. We are unfortunately unable to report a nonresponse rate due to methodology of dissemination. There was a discrepancy in number of intensivists compared to number of nephrologists that completed the survey (i.e., 140 to 39). While this ratio reflects the ratio of providers who maintain board certification in the respective specialties as discussed in the methods, the incongruity could skew the results. We also did not perform reliability testing to measure validity of our tool. Some components of the survey included medical information more specific to daily practice of intensivists (ex. vasoactive infusions or lung compliance) which may have skewed responses from nephrologists less familiar with these topics. Finally, our survey was disseminated via avenues that target physicians involved in American academic societies, and thus, their opinions and practices may differ from those in private practice or those in low resource or international settings. Survey respondents reported greater perceived personal emphasis on fluid balance than their institution which highlights the possibility that survey respondents are more interested in the topic at hand.

## 5 Conclusion

In conclusion, pediatric nephrologists and intensivists typically agree when it is appropriate to initiate fluid deresuscitation, except from early in shock. The way deresuscitation is accomplished had greater variability, especially regarding ultrafiltration and/or dialysis. This study highlights the necessity of widely accepted and operationalizable definitions, additional research, and eventual development of evidence-based consensus guidelines on deresuscitation strategies with the goal of improving patient outcomes.

## Data Availability

The raw data supporting the conclusions of this article will be made available by the authors, without undue reservation.
