# Supplementary figures and images for: Fluid deresuscitation in critically ill children: comparing perspectives of intensivists and nephrologists
Source: Front Pediatr. 2024 Oct 28;12:1484893. doi: 10.3389/fped.2024.1484893 (PMC11551605; doi:10.3389/fped.2024.1484893)

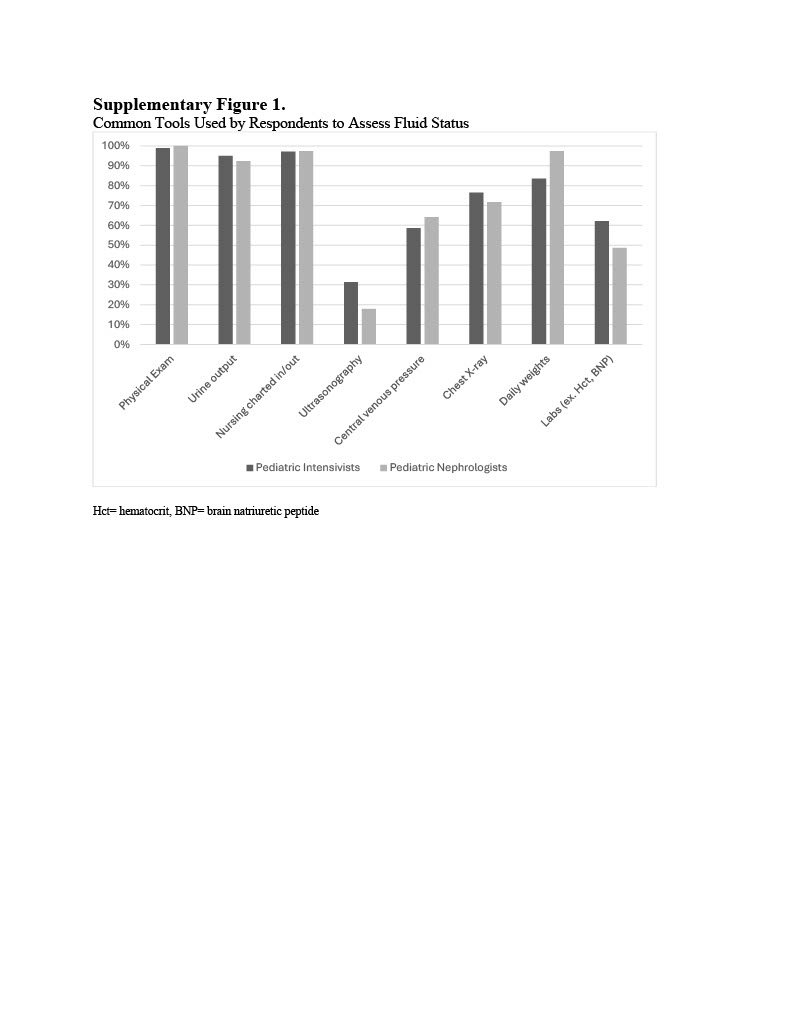

Supplement: Supplementary file 1 [file Image1.jpeg]
