# Supplementary material for: Fluid deresuscitation in critically ill children: comparing perspectives of intensivists and nephrologists
Source: Front Pediatr. 2024 Oct 28;12:1484893. doi: 10.3389/fped.2024.1484893 (PMC11551605; doi:10.3389/fped.2024.1484893)
Supplement: Supplementary file 2 [file Datasheet1.pdf]

Supplementary File 1.
Survey

Title of Research: Fluid De-resuscitation: Comparing the Approach of Intensivists and Nephrologists
UAB IRB Protocol #: IRB-300011177
Principal Investigator: Chloe Braun

Dear provider,
It is well established that positive fluid balance is associated with increased morbidity and mortality. Less well established is the optimal approach to fluid deresuscitation (Deresuscitation refers to the active removal of accumulated fluid using diuretics or ultrafiltration[1].). The aim of the FD-CAIN (Fluid Deresuscitation- Comparing Approach of Intensivists and Nephrologists) is to compare current practices among pediatric intensivists and pediatric nephrologists related to identifying markers of adequate resuscitation and strategies used in the transition to the de-resuscitation phase if/when indicated.
We invite you to participate by filling out the following survey. The estimated time requirement is 10 minutes. All responses are anonymous. By completing the survey you provide consent to participate in the study. Participation is voluntary. There are no known risks to participating in this survey. There will be no direct benefit to participating in this research. This project was approved by the IRB through the University of Alabama, Birmingham. You may contact the Principal Investigator, Chloe Braun, MD at cgmeyer@uabmc.edu with questions or comments.

Thank you for agreeing to participate and for your commitment to furthering clinical research.

If you have questions about your rights as a research participant, or concerns or complaints about the research, you may contact the UAB Office of the IRB (OIRB) at (205) 934-3789 or toll free at 1-855-860-3789. Regular hours for the OIRB are 8:00 a.m. to 5:00 p.m. CT, Monday through Friday.
Sincerely,
Chloe Braun

Do you provide consent to participate in this study?
Yes
No
Screening question: Are you a pediatric critical care physician or a pediatric nephrologist?
Yes—continue on with survey
No—survey end, do not meet requirements

---

1) In my personal practice, discussing fluid balance and initiating fluid de-resuscitation in ICU patients is:
   a) Very unimportant
   b) Moderately unimportant
   c) Neither important or unimportant
   d) Moderately important
   e) Very important
2) In general, at my institution, discussing fluid balance and initiating fluid de-resuscitation in ICU patients is:
   a) Very unimportant
   b) Moderately unimportant
   c) Neither important or unimportant
   d) Moderately important
   e) Very important
3) At my institution there is consistency among providers within my specialty in determining clinical stability prior to initiating fluid de-resuscitation.
   a) Strongly disagree
   b) Disagree
   c) Neither disagree or agree
   d) Agree
   e) Strongly agree
4) At my institution there is consistency among providers across multiple specialties in determining clinical stability prior to initiating fluid de-resuscitation.
   a) Strongly disagree
   b) Disagree
   c) Neither disagree or agree
   d) Agree
   e) Strongly agree
5) Does you institution follow a fluid de-resuscitation protocol for patients with positive fluid balance?
   a) Yes (Branching logic, if yes:, is the protocol triggered based off of a certain percent fluid overload? Yes/No)
      i) (Branching logic, If yes: What percent positive fluid balance triggers a response? __)
   b) No
6) Thinking about rounding on specific patients, how often is fluid balance explicitly discussed?
   a) All of the time
   b) Most of the time
   c) Some of the time
   d) Hardly ever
7) At your institution, when is the nephrologist involved with regard to fluid de-resuscitation? (Select all that apply)
   a) Always involved
   b) Involved only in complex cases (ex. Multi organ dysfunction, primary renal disease)
   c) Involved only if certain degree of fluid overload
   d) Rarely involved

8) Clinical vignette: 4-year-old male with ALL undergoing induction chemotherapy admitted for septic shock. Initial vital signs upon arrival to the PICU are HR 140, BP 68/39, T 103, RR 26, SpO2 97% on RA. Initial VBG revealed 7.1/45/-/7 with a lactate of 7. He receives 3x 20 cc/kg LR boluses and is started on IVF at 4-2-1 maintenance rate, norepinephrine at 0.05 mcg/kg/min and epinephrine at 0.05 mcg/kg/min (low dose) to maintain MAPs at 10th %ile for age.
   a) 2 hours after PICU admission his lactate has increased to 9.3. He receives 20 cc/kg pRBC for hematocrit of 19 and 20 cc/kg of platelets for a platelet count of 4,000. He is less responsive to access attempts and is sedated, muscle relaxed, and intubated for shock and depressed mental status. He receives 20 cc/kg LR prior to intubation. Norepinephrine is at 0.08 mcg/kg/min, epinephrine is at 0.06 mcg/kg/min (moderate dose).
      i) Patient is still in shock, de-resuscitation is inappropriate at this time
      ii) Patient is still in shock, but initiating de-resuscitation efforts is appropriate at this time
      iii) Patient is no longer in shock, but it is still too early to de-resuscitate patient
      iv) Patient is no longer in shock and de-resuscitation efforts are appropriate
   b) 12 hours after PICU admission lactate is 3.9. His vasopressor requirement and urine output remain unchanged. By ventilator mechanics his lung compliance is poor and he has crackles on physical exam.
      i) Patient is still in shock, de-resuscitation is inappropriate at this time
      ii) Patient is still in shock, but initiating de-resuscitation efforts is appropriate at this time
      iii) Patient is no longer in shock, but it is still too early to de-resuscitate patient
      iv) Patient is no longer in shock and de-resuscitation efforts are appropriate
   c) 24 hours after PICU admission lactate is 2.5. He remains on epinephrine at 0.02. Urine output has been about 0.5 cc/kg/h. His paralysis is discontinued. Blood cultures return positive for E.Coli. His mother thinks his eyes look puffy. CVP is 13.
      i) Patient is still in shock, de-resuscitation is inappropriate at this time
      ii) Patient is still in shock, but initiating de-resuscitation efforts is appropriate at this time
      iii) Patient is no longer in shock, but it is still too early to de-resuscitate patient
      iv) Patient is no longer in shock and de-resuscitation efforts are appropriate
   d) 48 hours after PICU admission lactate is 1.2. Primary team is considering extubation once lung mechanics improve. He is not on any vasoactive medications. Point of care ultrasound reveals small pleural effusions bilaterally. Labs reveal persistent evidence of end organ injury with mild transaminitis and his creatinine is double his baseline. Repeat blood cultures are no growth x24 hours.
      i) Patient is still in shock, de-resuscitation is inappropriate at this time
      ii) Patient is still in shock, but initiating de-resuscitation efforts is appropriate at this time
      iii) Patient is no longer in shock, but it is still too early to de-resuscitate patient
      iv) Patient is no longer in shock and de-resuscitation efforts are appropriate
9) What would be your next step? Choose all that apply. (If answered ii or iv on any of previous questions)
   a) Stop providing fluid boluses
   b) Decrease (or discontinue) maintenance IV fluids
   c) One time dose of loop diuretic with subsequent doses depending on response
   d) Intermittent loop diuretic scheduled
   e) Infusion of loop diuretic
   f) Dialysis/ultrafiltration
   g) Initial dose of loop diuretic followed by infusion
   h) Other: please specify
10) In general, how do you calculate fluid balance?
   a) Weight based
   b) In versus out based
   c) Both
   d) Neither
11) What tools do you commonly use to help assess patient fluid balance? (Select all that apply)
   a) Physical exam

b) Urine output
c) Nursing Charted ins/outs
d) Ultrasonography
e) Central venous pressure
f) Chest x-ray
g) Daily weights
h) Laboratory data (ex. Hematocrit, BNP)

12) In general, do you have the tools or means necessary to assess a patient's fluid balance?
    a) Yes (Branching logic, if yes: what tools or means would be necessary or wanted to better assess a patient's fluid balance? ___)
    b) No
13) In general, do you have the tools or means to accurately prescribe the fluid balance you want?
    a) Yes (Branching logic, if yes: what tools or means would be necessary or wanted to better prescribe a patient's fluid balance? ___)
    b) No

14) In critically ill children on mechanical ventilation, before starting deresuscitation, how do you determine fluid administration dosing?
    a) Intravenous fluids at the "4-2-1" rate in addition to other volume (example: blood products, medications or medication carriers)
    b) Goal total fluids at the 100% maintenance rate per the "4-2-1" rule including medications, feeds, IVF and carriers
    c) Total fluids (IVF, feeds, medications, carriers) at 75% of the maintenance rate
    d) Total fluids (IVF, feeds, medications, carriers) at 63% of the maintenance rate
    e) None of the above or patient dependent

15) After admission and stabilization (however defined), what is the typical timeframe when you would consider starting a diuretic for active fluid removal if a patient is not at their fluid balance goal:
    a) 0-24 hours
    b) 24-48 hours
    c) 48-72 hours
    d) Over 72 hours
    e) Only if evidence negative impact from fluid accumulation.
    f) None of the above or patient dependent

16) How does providing enteral nutrition impact your fluid management strategies? (Choose all that apply)
    a) I do not include enteral nutrition in the total in/out calculation
    b) I do include enteral nutrition in total in/out calculation
    c) I may lower the enteral feeding volume or increase caloric density of feeds to lower the volume to meet a fluid goal
    d) I do not alter feeding volume or caloric density of feeds
    e) None of the above

Demographic information

17) What is your specialty? (Select all that apply)
    a) Pediatric Nephrology
    b) Pediatric Critical Care
    c) Pediatric Cardiac Critical Care
18) How many years post-training (i.e. years following completion of fellowship)? ________

The following questions refer to the primary institution where you practice

19) Number of medical pediatric ICU beds (non-NICU) ___
20) What post-graduate trainees does your institution have? (Check all that apply)
    a) Pediatric residents
    b) Pediatric critical care fellows
    c) Pediatric nephrology fellows
    d) None

21) Does your institution have ECMO capabilities?
    a) Yes
    b) No
22) Does your institution have a specific cardiac ICU?
    a) Yes
    b) No
23) Does your institution have CRRT capabilities?
    a) Yes
        i) Best estimate, how many patients are on CRRT annually?
            (1) <10
            (2) 10-25
            (3) 25-50
            (4) >50
    b) No
24) What renal replacement capabilities do you have in the ICU? (Select all that apply)
    a) Peritoneal Dialysis
    b) Continuous renal replacement therapy
    c) Hemodialysis
    d) Prolonged intermittent renal replacement therapy
    e) Therapeutic Plasma Exchange
    f) Renal replacement therapy via extracorporeal membrane oxygenation

Abbreviations:

IVC- inferior vena cava
CVP- central venous pressure
ALL- acute lymphoblastic leukemia
E. Coli- Escherichia coli
HR- heart rate
BP- blood pressure
T- Temperature
RR- respiratory rate
SpO2- oxygen saturation
RA- room air
VBG- venous blood gas (pH/pCO2/pO2/pHCO3)
LA- lactic acid
LR- lactated ringers
IVF- intravenous fluid
MAPs- mean arterial pressure
CRRT- continuous renal replacement therapy
FO- fluid overload
pRBC- packed red blood cells

1. Silversides, J. A., McMullan, R., Emerson, L. M., Bradbury, I., Bannard-Smith, J., Szakmany, T., Trinder, J., Rostron, A. J., Johnston, P., Ferguson, A. J., Boyle, A. J., Blackwood, B., Marshall, J. C., & McAuley, D. F. (2022). Feasibility of conservative fluid administration and deresuscitation compared with usual care in critical illness: the Role of Active Deresuscitation After Resuscitation-2 (RADAR-2) randomised clinical trial. Intensive care medicine, 48(2), 190–200. https://doi.org/10.1007/s00134-021-06596-8
