# Supplementary material for: Fluid deresuscitation in critically ill children: comparing perspectives of intensivists and nephrologists
Source: Front Pediatr. 2024 Oct 28;12:1484893. doi: 10.3389/fped.2024.1484893 (PMC11551605; doi:10.3389/fped.2024.1484893)
Supplement: Supplementary file 3 [file Datasheet2.pdf]

Supplementary Table 1.

Deresuscitation Initiation by Number of Pediatric ICU Beds

Comparison of intensivists' to nephrologists' responses to whether the clinical vignette patient is (1) in shock or not in shock and (2) initiation of deresuscitation is appropriate or inappropriate. Time points refer to each stage of ROSE framework. See Figure 1 for framework and timepoint descriptions. Responses were split into two subgroups: number of ICU beds ≥26 beds (left three columns) or <26 beds (right three columns). Cut off of 26 beds was based on median self-reported number of beds.

*Missing data from two nephrologists excluded due to unknown number of PICU beds.

Answer option	**≥26 PICU beds**			**<26 PICU beds**		
	Intensivists	Nephrologists	P value	Intensivists	Nephrologists	P value
**Case time point 1: Resuscitation Phase**						
Patient is in **shock**<br>Deresuscitation is **appropriate**	5.6%<br>(4/72)	26.3%<br>(5/19)	<0.01	7.4%<br>(5/68)	11.1%<br>(2/18)	0.62
Patient is in **shock**<br>Deresuscitation is **inappropriate**	93.1%<br>(67/72)	63.2%<br>(12/19)		92.7%<br>(63/68)	88.9%<br>(16/18)	
Patient is **not in shock**<br>Deresuscitation is **appropriate**	0%	0%		0%	0%	
Patient is **not in shock**<br>Deresuscitation is **inappropriate**	1.4%<br>(1/72)	10.5%<br>(2/19)		0%	0%	
**Case time point 2: Optimization Phase**						
Patient is in **shock**<br>Deresuscitation is **appropriate**	51.4%<br>(37/72)	47.4%<br>(9/19)	0.04	61.8%<br>(42/68)	55.5%<br>(10/18)	0.61
Patient is in **shock**<br>Deresuscitation is **inappropriate**	34.7%<br>(25/72)	15.8%<br>(3/19)		17.6%<br>(12/68)	11.1%<br>(2/18)	
Patient is **not in shock**<br>Deresuscitation is **appropriate**	6.9%<br>(5/72)	31.6%<br>(6/19)		8.8%<br>(6/68)	16.7%<br>(3/18)	
Patient is **not in shock**<br>Deresuscitation is **inappropriate**	6.9%<br>(5/72)	5.3%<br>(1/19)		11.7%<br>(8/68)	16.7%<br>(3/18)	
**Case time point 3: Stabilization Phase**						
Patient is in **shock**<br>Deresuscitation is **appropriate**	38.9%<br>(28/72)	26.3%<br>(5/19)	0.34	27.9%<br>(19/68)	27.8%<br>(5/18)	0.36
Patient is in **shock**<br>Deresuscitation is **inappropriate**	2.8%<br>(2/72)	0%		0%	5.6%<br>(1/18)	
Patient is **not in shock**<br>Deresuscitation is **appropriate**	55.6%<br>(40/72)	73.7%<br>(14/19)		66.2%<br>(45/68)	61.1%<br>(11/18)	
Patient is **not in shock**<br>Deresuscitation is **inappropriate**	2.8%<br>(2/72)	0%		5.9%<br>(4/68)	5.6%<br>(1/18)	
**Case time point 4: Evacuation Phase**						
Patient is in **shock**<br>Deresuscitation is **appropriate**	1.4%<br>(1/72)	0%	0.38	1.5%<br>(1/68)	0%	0.38
Patient is in **shock**<br>Deresuscitation is **inappropriate**	0%	0%		0%	0%	
Patient is **not in shock**<br>Deresuscitation is **appropriate**	94.4%<br>(68/72)	100%<br>(19/19)		94.1%<br>(64/68)	100%<br>(18/18)	
Patient is **not in shock**<br>Deresuscitation is **inappropriate**	4.2%<br>(3/72)	0%		4.4%<br>(3/68)	0%	

Supplementary Table 2.

Deresuscitation Initiation by Years of Experience

Comparison of intensivists' to nephrologists' responses to whether the clinical vignette patient is (1) in shock or not in shock and (2) initiation of deresuscitation is appropriate or inappropriate. Time points refer to each stage of ROSE framework. See Figure 1 for framework and timepoint descriptions. Responses were split into two subgroups: ≥7 years of post-training experience (left three columns) or <7 years of post-training experience (right three columns). Cut off of 7 years was based on median self-reported number of years.

Answer option	**≥7 years of experience**			**<7 years of experience**		
	Intensivists	Nephrologists	P value	Intensivists	Nephrologists	P value
Case time point 1: Resuscitation Phase						
Patient is in **shock**<br>Deresuscitation is **appropriate**	6.7% (5/75)	16.7% (4/24)	0.08	6.2% (4/65)	20.0% (3/15)	0.16
Patient is in **shock**<br>Deresuscitation is **inappropriate**	93.3% (70/75)	79.2% (19/24)		92.3% (60/65)	73.3% (11/15)	
Patient is **not in shock**<br>Deresuscitation is **appropriate**	0%	0%		0%	0%	
Patient is **not in shock**<br>Deresuscitation is **inappropriate**	0%	4.2% (1/24)		1.5% (1/65)	6.7% (1/15)	
Case time point 2: Optimization Phase						
Patient is in **shock**<br>Deresuscitation is **appropriate**	48.0% (36/75)	50.0% (12/24)	0.05	66.2% (43/65)	53.3% (8/15)	0.66
Patient is in **shock**<br>Deresuscitation is **inappropriate**	29.3% (22/75)	8.3% (2/24)		23.1% (15/65)	26.7% (4/15)	
Patient is **not in shock**<br>Deresuscitation is **appropriate**	10.7% (8/75)	29.2% (7/24)		4.6% (3/65)	13.3% (2/15)	
Patient is **not in shock**<br>Deresuscitation is **inappropriate**	12.0% (9/75)	12.5% (3/24)		6.2% (4/65)	6.7% (1/15)	
Case time point 3: Stabilization Phase						
Patient is in **shock**<br>Deresuscitation is **appropriate**	26.7% (20/75)	29.2% (7/24)	0.85	41.5% (27/65)	20.0% (3/15)	0.13
Patient is in **shock**<br>Deresuscitation is **inappropriate**	1.3% (1/75)	0%		1.5% (1/65)	13.3% (2/15)	
Patient is **not in shock**<br>Deresuscitation is **appropriate**	65.3% (49/75)	66.7% (16/24)		55.3% (36/65)	66.7% (10/15)	
Patient is **not in shock**<br>Deresuscitation is **inappropriate**	6.7% (5/75)	4.2% (1/24)		1.5% (1/65)	0%	
Case time point 4: Evacuation Phase						
Patient is in **shock**<br>Deresuscitation is **appropriate**	1.3% (1/75)	0%	0.32	1.5% (1/65)	0%	0.43
Patient is in **shock**<br>Deresuscitation is **inappropriate**	0%	0%		0%	0%	
Patient is **not in shock**<br>Deresuscitation is **appropriate**	94.7% (71/75)	100% (24/24)		93.8% (61/65)	100% (15/15)	
Patient is **not in shock**<br>Deresuscitation is **inappropriate**	4.0% (3/75)	0%		4.6% (3/65)	0%	

Supplementary Table 3.

Deresuscitation Initiation among Nephrologists Subgroups

From the nephrology cohort only, comparison of responses to whether the clinical vignette patient is (1) in shock or not in shock and (2) initiation of deresuscitation is appropriate or inappropriate. Time points refer to each stage of ROSE framework. See Figure 1 for framework and timepoint descriptions. This table compares nephrologists who self-reported ≥26 bed PICU or <26 bed PICU in left three columns and self-reported ≥7 years of experience or <7 years of experience in right three columns. Subgroups were split into ≥26 beds or <26 beds and ≥7 years and <7 years based on median self-reported number of beds/years of experience.

*Missing data from two nephrologists excluded due to unknown number of PICU beds.

**Nephrologists Only**						
Answer option	≥26 Beds	<26 beds	P value	≥ 7 years	<7 years	P value
Case time point 1: Resuscitation Phase						
Patient is in **shock**<br>Deresuscitation is **appropriate**	26.3% (5/19)	11.1% (2/18)	0.15	16.7% (4/24)	20.0% (3/15)	0.90
Patient is in **shock**<br>Deresuscitation is **inappropriate**	63.2% (12/19)	88.9% (16/18)		79.2% (19/24)	73.3% (11/15)	
Patient is **not in shock**<br>Deresuscitation is **appropriate**	0%	0%		0%	0%	
Patient is **not in shock**<br>Deresuscitation is **inappropriate**	10.5% (2/19)	0%		4.2% (1/24)	6.7% (1/15)	
Case time point 2: Optimization Phase						
Patient is in **shock**<br>Deresuscitation is **appropriate**	47.4% (9/19)	55.6% (10/18)	0.53	50.0% (12/24)	53.3% (8/15)	0.34
Patient is in **shock**<br>Deresuscitation is **inappropriate**	15.8% (3/19)	11.1% (3/18)		8.3% (2/24)	26.7% (4/15)	
Patient is **not in shock**<br>Deresuscitation is **appropriate**	31.6% (6/19)	16.7% (3/18)		29.2% (7/24)	13.3% (2/15)	
Patient is **not in shock**<br>Deresuscitation is **inappropriate**	5.3% (1/19)	16.7% (3/18)		12.5% (3/24)	6.7% (1/15)	
Case time point 3: Stabilization Phase						
Patient is in **shock**<br>Deresuscitation is **appropriate**	26.3% (5/19)	27.8% (5/18)	0.51	29.1% (7/24)	20.0% (3/15)	0.25
Patient is in **shock**<br>Deresuscitation is **inappropriate**	0%	5.6% (1/18)		0%	13.3% (2/15)	
Patient is **not in shock**<br>Deresuscitation is **appropriate**	73.7% (14/19)	61.1% (11/18)		66.7% (16/24)	66.7% (10/15)	
Patient is **not in shock**<br>Deresuscitation is **inappropriate**	0%	5.6% (1/18)		4.2% (1/24)	0%	
Case timepoint 4: Evacuation Phase						
Patient is in **shock**<br>Deresuscitation is **appropriate**	0%	0%	1.0	0%	0%	1.0
Patient is in **shock**<br>Deresuscitation is **inappropriate**	0%	0%		0%	0%	
Patient is **not in shock**<br>Deresuscitation is **appropriate**	100% (19/19)	100% (18/18)		100% (24/24)	100% (15/15)	
Patient is **not in shock**<br>Deresuscitation is **inappropriate**	0%	0%		0%	0%	

Supplementary Table 4.

Deresuscitation Initiation among Intensivists Subgroups

From the intensivist cohort only, comparison of responses to whether the clinical vignette patient is (1) in shock or not in shock and (2) initiation of deresuscitation is appropriate or inappropriate. Time points refer to each stage of ROSE framework. See Figure 1 for framework and timepoint descriptions. This table compares intensivists who self-reported ≥26 bed PICU or <26 bed PICU in left three columns and self-reported ≥7 years of experience or <7 years of experience in right three columns. Subgroups were split into ≥26 beds or <26 beds and ≥7 years and <7 years based on median self-reported number of beds/years of experience.

**Intensivists Only**						
Answer option	≥26 Beds	<26 beds	P value	≥ 7 years	<7 years	P value
Case time point 1: Resuscitation Phase						
Patient is in **shock** Deresuscitation is **appropriate**	5.6% (4/72)	7.4% (5/68)	0.57	6.7% (5/75)	6.2% (4/65)	0.46
Patient is in **shock** Deresuscitation is **inappropriate**	93.1% (67/72)	92.7% (63/68)		93.3% (70/75)	92.3% (60/65)	
Patient is **not in shock** Deresuscitation is **appropriate**	0%	0%		0%	0%	
Patient is **not in shock** Deresuscitation is **inappropriate**	1.4% (1/72)	0%		0%	1.5% (1/65)	
Case time point 2: Optimization Phase						
Patient is in **shock** Deresuscitation is **appropriate**	51.4% (37/72)	61.8% (42/68)	0.14	48.0% (36/75)	66.2% (43/65)	0.13
Patient is in **shock** Deresuscitation is **inappropriate**	34.7% (25/72)	17.6% (12/68)		29.3% (22/75)	23.1% (15/65)	
Patient is **not in shock** Deresuscitation is **appropriate**	6.9% (5/72)	8.8% (6/68)		10.7% (8/75)	4.6% (3/65)	
Patient is **not in shock** Deresuscitation is **inappropriate**	6.9% (5/72)	11.8% (8/68)		12.0% (9/75)	6.2% (4/65)	
Case time point 3: Stabilization Phase						
Patient is in **shock** Deresuscitation is **appropriate**	38.9% (28/72)	27.9% (19/68)	0.21	26.7% (20/75)	41.5% (27/65)	0.16
Patient is in **shock** Deresuscitation is **inappropriate**	2.8% (2/72)	0%		1.3% (1/75)	1.5% (1/65)	
Patient is **not in shock** Deresuscitation is **appropriate**	55.6% (40/72)	66.2% (45/68)		65.3% (49/75)	55.4% (36/65)	
Patient is **not in shock** Deresuscitation is **inappropriate**	2.8% (2/72)	5.9% (4/68)		6.7% (5/75)	1.5% (1/65)	
Case timepoint 4: Evacuation Phase						
Patient is in **shock** Deresuscitation is **appropriate**	1.4% (1/72)	1.5% (1/68)	1.0	1.3% (1/75)	1.5% (1/65)	0.98
Patient is in **shock** Deresuscitation is **inappropriate**	0%	0%		0%	0%	
Patient is **not in shock** Deresuscitation is **appropriate**	94.4% (68/72)	94.1% (64/68)		94.7% (71/75)	93.9% (61/65)	
Patient is **not in shock** Deresuscitation is **inappropriate**	4.2% (3/72)	4.4% (3/68)		4.0% (3/75)	4.6% (3/65)	
